# The human cathelicidin peptide LL-37 inhibits pancreatic cancer growth by suppressing autophagy and reprogramming of the tumor immune microenvironment

**DOI:** 10.3389/fphar.2022.906625

**Published:** 2022-07-22

**Authors:** Zhu Zhang, Wen-Qing Chen, Shi-Qing Zhang, Jing-Xuan Bai, Ching-Lam Lau, Stephen Cho-Wing Sze, Ken Kin-Lam Yung, Joshua Ka-Shun Ko

**Affiliations:** ^1^ Teaching and Research Division, School of Chinese Medicine, Hong Kong Baptist University, Hong Kong SAR, China; ^2^ Department of Biology, Hong Kong Baptist University, Hong Kong SAR, China; ^3^ Golden Meditech Centre for NeuroRegeneration Sciences, Hong Kong Baptist University, Hong Kong SAR, China; ^4^ JNU-HKUST Joint Laboratory for Neuroscience and Innovative Drug Research, College of Pharmacy, Jinan University, Guangzhou, China; ^5^ Centre for Cancer and Inflammation Research, School of Chinese Medicine, Hong Kong Baptist University, Hong Kong SAR, China

**Keywords:** pancreatic cancer, LL-37, autophagy, ROS, mTOR signaling, tumor immune microenvironment

## Abstract

Pancreatic cancer is amongst the most lethal malignancies, while its poor prognosis could be associated with promotion of autophagy and the tumor immune microenvironment. Studies have confirmed the pro-tumorigenic nature of the cathelicidin family of peptide LL-37 in several types of cancer. However, at higher doses, LL-37 exerts significant cytotoxicity against gastrointestinal cancer cells. In our study, we investigated the anti-tumorigenic potential of LL-37 in pancreatic cancer and the underlying mechanisms. Our results have shown that LL-37 inhibited the growth of pancreatic cancer both *in vitro* and *in vivo*. Mechanistic studies have demonstrated that LL-37 induced DNA damage and cell cycle arrest through induction of reactive oxygen species (ROS). Further study indicates that LL-37 suppressed autophagy in pancreatic cancer cells through activation of mTOR signaling, leading to more accumulation of ROS production and induction of mitochondrial dysfunctions. With combined treatment of LL-37 with the mTOR inhibitor rapamycin, LL-37-induced ROS production and cancer cell growth inhibition were attenuated. Subsequent *in vivo* study has shown that LL-37 downregulated the immunosuppressive myeloid-derived suppressor cells and M2 macrophages while upregulated the anti-cancer effectors CD8^+^ and CD4^+^ T cells in the tumor microenvironment. By using an *in vitro* co-culture system, it was shown that promotion of M2 macrophage polarization would be suppressed by LL-37 with inhibition of autophagy, which possessed significant negative impact on cancer growth. Taken together, our findings implicate that LL-37 could attenuate the development of pancreatic cancer by suppressing autophagy and reprogramming of the tumor immune microenvironment.

## Introduction

Pancreatic cancer, including its most prominent type pancreatic ductal adenocarcinoma (PDAC), is one of the most annihilating neoplasms with very low survival rate. According to recent reports, among 495,773 new cases of PDAC worldwide in 2020, there were 466,003 related deaths (Sung et al., 2021). In contrast to the increasing survival rates of patients of other cancer types, the 5-years survival rate of pancreatic cancer patients is only 3%, while the associated mortality rate has been progressively increased by 0.3% annually among male patients (Siegel et al., 2018). Surgical resection of localized solid tumors is the key potential treatment for PDAC. However, this malignancy often has a late diagnosis and indicated to be resectable in approximately 15% of cases (Skrypek et al., 2013). For non-resectable advanced-stage PDAC, gemcitabine remains the mainstream chemotherapeutic drug and is the gold standard for locally advanced and metastatic PDAC to relieve symptoms and slightly improve overall survival (Springfeld et al., 2019). Unfortunately, most patients have developed resistance to gemcitabine within weeks of initiatial treatment, thus results in poor overall survival (Kim and Gallick 2008). The late diagnosis, poor prognosis, ineffective treatment approach and early metastasis all implicate the urge to identify neoadjuvant agents that could facilitate target-oriented mechanistic control of pancreatic cancer.

Autophagy is a highly conserved cellular process that involves intracellular degradation of cytoplasmic constituents including misfolded proteins, intracellular aggregates and damaged organelles (Yang and Klionsky 2010). Although autophagy is generally considered as a survival mechanism, in tumor cells this process plays a dual role by regulating both tumor cell death and survival. As a matter of fact, autophagy suppresses solid tumor growth by eliminating damaged organelles and recycling macromolecules during the process of benign-to-malignant transformation and early carcinogenesis in general (Mathew et al., 2007; Mathew et al., 2009). To the contrary, increasing evidence has also suggested that autophagy may facilitate tumorigenesis in response to metabolic stressors such as nutrient deprivation, hypoxia, growth factor depletion and anticancer therapy-induced stress in differentiated and advanced tumors (Janku et al., 2011; Maycotte and Thorburn, 2011). When compared with other malignancies, such as lung and breast cancers, a higher basal level of autophagy has been observed in pancreatic cancer cells and tumor tissues, which was determined to correlate with the poor prognosis in patients (Fujii et al., 2008; Yang et al., 2011). Increased autophagy has been detected in most pancreatic intraepithelial neoplasm-3 (PanIN-3) tissues and nearly all PDAC cell lines, causing attenuation of reactive oxygen species (ROS), mitigation of DNA damage and accumulation of tricarboxylic acid cycle intermediates to maintain energy homeostasis, which all contribute to tumor growth promotion (Yang and Kimmelman, 2011). Under such condition, pharmacological inhibition of autophagy by drugs such as chloroquine (CQ) and its derivative hydroxychloroquine (HCQ), or genetic knockdown using RNA interference to alter the expression of multiple key autophagy genes could be effective treatment strategies. However, current clinical applications of CQ and HCQ remain limited because of their ocular toxicity and weak single-agent anticancer efficacy (Sui et al., 2013). In addition, these drugs prompt serious collateral side effects on other cellular processes, whereas the impaired tumor metabolism induced by autophagy inhibition may interrupt the *in situ* antitumor immune response in the tumor microenvironment (Townsend et al., 2012). CQ and HCQ have been determined to impair inflammatory immune responses by suppressing macrophage-associated antigen and calcium signals in T cells (Ziegler and Unanue, 1982; Goldman et al., 2000). Furthermore, tumor cells with *Atg5* or *Atg7* knockdown fail to elicit CD4^+^ and CD8^+^ T cell activities and deactivate the natural protective antitumor immune response (Michaud et al., 2011). Therefore, new strategies to target autophagy must rely on better understanding of the tumor cell immunity.

LL-37 is a 37-amino acid C-terminal peptide (CAMP, hCAP18) of the only human cathelicidin protein (Larrick et al., 1995). LL-37 possesses antibacterial, antifungal, antiviral and wound healing functions (Vandamme et al., 2012). However, studies on the role of LL-37 in cancers have controversial results. At a concentration of 5 μg/ml, LL-37 exerts pro-tumorigenic effects in ovarian (Coffelt et al., 2008), lung (von Haussen et al., 2008), breast (Weber et al., 2009; Hensel et al., 2011), prostate cancers (Hensel et al., 2011), malignant melanoma (Jia et al., 2017) and skin squamous cell carcinoma (Wang et al., 2017). At higher concentrations, however, LL-37 induces anti-cancer effects in colon cancer (Ren et al., 2012; Kuroda et al., 2017), gastric cancer (Wu et al., 2010), hematologic malignancies (Mader et al., 2009) and oral squamous cell carcinoma (Chen et al., 2017). Although low concentrations of LL-37 were shown to promote pancreatic cancer stem cell-mediated tumorigenesis (Sainz et al., 2015), the effects of using higher doses of LL-37 on pancreatic cancer cells remain unclear.

In this study, we have demonstrated for the first time the growth-inhibitory effects of LL-37 in PDAC both *in vitro* and *in vivo*. Further mechanistic study has suggested that inhibition of cytoprotective autophagy and reprogramming of the tumor immune microenvironment by LL-37 are responsible for its anticancer potential in pancreatic cancer.

## Materials and methods

### Cell culture and chemicals

The human pancreatic carcinoma cell lines PANC1 and MIA PaCa-2 isolated from PDAC cell origin as well as the BALB/c mice-derived macrophages RAW264.7 were purchased from American Type Culture Collection (ATCC, Manassas, VA, United States). The murine pancreatic adenocarcinoma cell line Pan02 was kindly provided by Prof. Cho of The Chinese University of Hong Kong. Cells were grown in Dulbecco’s modified Eagle’s medium supplemented with 10% fetal bovine serum plus 1% penicillin and streptomycin, incubated at 37°C with a 5% CO_2_ atmosphere. All culture reagents were purchased from Thermo Fisher Scientific (Waltham, MA, United States). LL-37 (#LL37-002, ≥95% purity) was purchased from Chinese Peptide Company (Hangzhou, China). N-acetyl cysteine (NAC, # A9165-5G) and rapamycin (#R0395, ≥95% purity) were purchased from Sigma-Aldrich (St. Louis, MO, United States). For Western immunoblotting, primary antibodies specific for the following proteins were purchased from Cell Signaling Technology (Danvers, MA, United States): Beclin-1 (#3738S), Atg5 (#12994), LC3B (#2775S), phosphorylated (p)-AMPKα (#2534), AMPKα (#2532), p-ERK1/2 (#2470S), ERK1/2 (#4695S), p-mTOR (#5536S), mTOR (#2983S), γH2AX (#9718), CDK4 (#2546S), CDK6 (#3136), cdc2 (#77055S), CyclinD1 (#2978T), Cyclin B1 (#4138S), CD206 (#24595), Arg1 (#93668T) and *ß*-actin (#4967). The following secondary antibodies were purchased from Bio-Rad Laboratories (Hercules, CA, United States): horseradish peroxidase (HRP)-conjugated goat anti-mouse IgG (#1706516) and HRP-conjugated goat anti-rabbit IgG (#1706516). For the immunofluorescence assay, primary antibodies specific for the following proteins were purchased from BD Biosciences (San Jose, CA, United States): Gr-1 (#562709), CD3 (#565642), and F4/80 (#565635). CD11b (#17800), CD4 (#93518), CD8 (#55397) and CD206 (#24595) were purchased from Cell Signaling Technology. The following fluorescence-conjugated secondary antibodies were purchased from Thermo Fisher Scientific: ALexa Fluor 488-conjugated goat anti-rabbit IgG (H + L) cross-adsorbed secondary antibody (#A32731), ALexa Fluor 488-conjugated Goat anti-Mouse IgG (H + L) Cross-Adsorbed Secondary Antibody (#A11001), ALexa Fluor 488-conjugated Goat anti-Rat IgG (H + L) Cross-Adsorbed Secondary Antibody (#A11006), ALexa Fluor 633-conjugated Goat anti-Rabbit IgG (H + L) Cross-Adsorbed Secondary Antibody (#A21070), ALexa Fluor 633-conjugated Goat anti-Mouse IgG (H + L) Cross-Adsorbed Secondary Antibody (#A21050). For the flow cytometry assay of macrophages subtypes, antibodies were purchased from Biolegend (San Diego, CA, United States): F4/80 (#123120), CD86 (#105014) and CD206 (#141706).

### Cell viability test

Cell viability was measured using the standard 3-(4,5-dimethylthiazol-2-yl)-2,5-diphenyl-tetrazolium bromide (MTT) assay. PANC1 and MIA PaCa-2 cells were seeded at densities of 4.0×10^3^/well and 6.0×10^3^/well, respectively, into 96-well plates and allowed to adhere overnight. Cells were treated with different concentrations of LL-37 (1–32 μM) for 24–72 h and then incubated with 2.5% MTT solution (5 mg/ml) for another 3 h at 37°C. Dimethylsulfoxide (DMSO) was then used as to dissolve the formazan crystals produced before spectrophotometric analysis at 570 nm (absorbance) and 650 nm (reference). The percentage of cell viability was calculated as the percentage change in the absorbance of LL-37-treated cells divided by the absorbance of DMSO-treated cells.

### Colony formation assay

PDAC cells were treated with LL-37 (0, 8 and 16 μM) for 24 h in 6-well plates (0.7 × 10^3^ cells/well) and incubated in DMEM supplemented with 10% FBS plus 1% penicillin and streptomycin. The medium was replaced every 3 days. After culturing for 12 days, the colonies were washed with PBS, fixed in 4% paraformaldehyde (PFA) for 20 min, stained with 0.1% crystal violet for 30 min and washed with PBS. The number of colonies and the colony number ratio were calculated using the ImageJ software.

### 5-Ethynyl-2′-deoxyuridine proliferation assay (EdU)

The effect of LL-37 on cell proliferation was determined by using a Cell-Light™ EdU Apollo^®^567 *In Vitro* Imaging Kit (#C10371-1, Ribobio, Guangzhou, China) according to the manufacture’s instruction. In brief, following LL-37 treatment, cells were incubated with EdU (100 μM) for 2 h followed by a series of other processes. EdU-stained images were captured under a fluorescent microscope (Nikon, Japan). At least three independent experiments had been conducted with replicates, and three visual fields per well were randomly selected for quantitative analysis.

### Immunofluorescence assay

After treatment with LL-37 (0, 8, 16 µM) for 24 h, PANC1 and MIA PaCa-2 cells were washed with phosphate-buffered saline and fixed in 4% paraformaldehyde. After permeabilization with 0.5% Triton X-100 and blocking in 5% bovine serum albumin (BSA), cells were incubated with primary antibodies specific for LC3B and γ-H2AX overnight at 4°C, followed by fluorescence-conjugated secondary antibodies at room temperature for 1 h. The nuclei were stained with 4′,6-diamidino-2-phenylindole (DAPI) for 5 min. Pseudocolor images were obtained using a Zeiss Instruments confocal microscope (Carl Zeiss, Oberkochen, Germany).

### Western blot analysis

Cells were treated with LL-37 (0, 8, 16 µM) and lysed in radioimmunoprecipitation buffer containing 50 mM Tris, 150 mM NaCl, 0.5% deoxycholate, 0.1% sodium dodecyl sulfate, 2 mM ethylenediaminetetraacetic acid, 0.1% Triton X-100, 10% glycerol, 1 mM phenylmethylsulfonyl fluoride and 10 μg/ml aprotinin. The Coomassie Plus Protein Assay Reagent kit (#23238, Thermo Fisher Scientific) was used to quantify the proteins in the cell lysates. The total cellular proteins in the lysates were then separated by 8–15% sodium dodecyl sulfate polyacrylamide gel electrophoresis and transferred to nitrocellulose membranes. After conjugation with primary and secondary antibodies, immunoreactive bands were visualized using a chemiluminescence substrate. The intensity of each band was quantified using ImageJ software (National Institutes of Health, Bethesda, MD, United States) and normalized to *ß*-actin housekeeping control.

### Cell cycle analysis

After LL-37 (0, 8, 16 µM) treatment for 24 h, PANC1 and MIA PaCa-2 cells were harvested and fixed with ice-cold 70% ethanol at −20°C overnight. The FxCycle™ PI/RNase Kit was used to stain the DNA according to the manufacturer’s instructions. Samples were analyzed by flow cytometry (FACS Canto™, Beckton Dickinson Biosciences, San Jose, CA, United States), and the percentages of cells at different phases of the cell cycle were calculated using ModFitLT version 3.0 software (BD Biosciences, San Jose, CA, United States).

### Autophagy flux sensor

Autophagic flux was monitored by using the Premo™ Autophagy Tandem Sensor RFP-GFP-LC3B Kit (#P36239, Thermo Scientific) according to the manufacturer’s instructions. Twelve μL of tandem LC3II-fluorescent protein was added to 4 × 10^4^ cells and incubated for 24 h. Following LL-37 treatment for another 24 h, the resulting images were analyzed using a Zeiss Instruments confocal microscope.

### Transmission electron microscopy (TEM)

After LL-37 treatment for 24 h, cells were fixed in TEM stationary solution (2.5% glutaraldehyde in 0.2 M HEPES, #G1102, Servicebio Technology) at 4°C for 4 h, rinsed in PBS, and then embedded in 4% agarose. After fixation in 1% osmium tetroxide for 2 h, specimens were dehydrated using alcohol and embedded in polybed 812 resin. After polymerization at 60°C for 48 h, ultra-thin sections were prepared with the Leica Ultracutuct slicer (Leica, Germany), stained with uranyl acetate and lead citrate, and finally analyzed using TEM (HITACHI).

### Measurement of intracellular ROS

After treatment with corresponding agents for 24 h, PANC1 and MIA PaCa-2 cells were stained by the fluorescence dye CM-H2DCFDA (#C6827, Thermo Fisher Scientific) and analyzed using immunofluorescence microscopy according to the manufacturer’s instructions. The ROS fluorescence intensity was measured using a SpectraMax M2 Plate Reader (Molecular Devices) with excitation/emission wavelengths of 495/520 nm.

### Mitochondrial membrane potential assay (MMP)

Mitochondrial membrane potential was measured using the JC-1 mitochondrial membrane potential probe (#T3168, Invitrogen). After treatment with LL-37, PANC1 and MIA PaCa-2 cells were incubated for 20 min with 2 μg/ml of JC-1 in culture medium at 37°C. After washing with PBS three times, the cells were imaged using a Zeiss Instruments confocal microscope. The red/green fluorescence ratio was quantified by the ImageJ software.

### Animal study

Six-week-old male C57/BL6 mice were purchased from the Laboratory Animal Services Centre of The Chinese University of Hong Kong. The animal experimentation protocols were carried out with the prior approval by the Research Ethics Committee of Hong Kong Baptist University (REC/18-19/0184) and according to the Animals (Control of Experiments) Regulations of the Department of Health, Hong Kong SAR, China (Licence no. (18-112) in DH/SHS/8/2/6 Pt.2). The tumor xenograft model was established by subcutaneously inoculating Pan02 cells (10^6^ cells per mouse) into the right flank of each mouse. After 6 days, mice were randomly divided into the following four groups (*n* = 6): Control (vehicle) group, chemotherapeutic drug group (gemcitabine: 50 mg/kg), low-dose LL-37 group (10 mg/kg) and high-dose LL-37 group (20 mg/kg). All drugs were prepared in 5% DMSO/30% PEG-400/5% Tween-80. Mice received various drug treatments for 14 days (by daily intraperitoneal injection of vehicle or LL-37, or administration of gemcitabine every 3 days). The body weights and tumor diameters were measured every 3 days, while the tumor volume was calculated using the formula: (short diameter)^2^ × (long diameter) × 0.5. At the end of the experiments, peripheral blood was collected from the orbital plexus for the detection of white blood cells and hematocrit. The excised tumors were weighed and prepared for subsequent histological study by fixing in 10% formalin and embedded in paraffin. Next, the tumor specimens were sectioned for TUNEL assay and immunohistochemical (IHC) analysis.

### Immunofluorescence of animal tissues

Paraffin-embedded tissue sections from the excised tumors were subject to immunofluorescence analysis as previously described (Porembka et al., 2012). Antibodies specific for CD11b, Gr-1, CD3, CD4, CD8, CD206 and F4/80 were used to determine the co-localization of myeloid-derived suppressor cells (MDSCs), CD4^+^ and CD8^+^ T cells as well as M2 macrophages, respectively.

### Effect on the polarization of M2 macrophages

The effect of LL-37 on M2 macrophage polarization was determined as described in our previous study, with slight modifications (Zhang et al., 2020). The confluent RAW264.7 cells at a density of 1 × 10^6^/well in 6-well plates were cultured in serum-free medium to induce growth arrest, designated as M0 macrophages. The M0 macrophages were polarized to M1 macrophages by adding 100 ng/ml LPS (#tlrl-eblps, InvivoGen) or to M2 macrophages by adding 20 ng/ml of mouse recombinant IL-4 (#214-14, PeproTech). To study the effect of LL-37 on M2 polarization, M0 macrophages were treated by LL-37 at a concentration not inducing growth inhibition (1 μM) for 12 h, followed by treatment with 20 ng/ml of IL-4, which was designated as M2+LL-37 macrophages. The expression of the cell surface markers F4/80, CD86 and CD206 were used to determine the macrophage subtypes by using a flow cytometer. And the cells were also harvested for protein expression analysis of the M2 markers CD206 and Arg1 using Western immunoblotting.

To evaluate the effect of LL-37-regulated M2 macrophage polarization on pancreatic cancer cell growth, macrophage-conditioned medium was prepared. The confluent RAW264.7 cells were prepared as described above. After washing with PBS, these macrophages were cultured in serum-free medium for another 24 h. Conditioned medium was finally obtained from the supernatant after centrifugation. The conditioned medium from the M0, M1, M2 and M2+LL-37 macrophages were designated as CM-M0, CM-M1, CM-M2, and CM-M2+LL-37, respectively. PAN02 cells were cultured with these conditioned media and Dulbecco’s modified Eagle’s medium supplemented with 10% fetal bovine serum DMEM (volume ratio 4:1) for 24 h, after which cell viability was tested by using the MTT assay.

### Statistical analysis

Results are presented as means ± standard deviations (SD) from at least three replicates. Statistical significance at a level of *p* < 0.05 was determined using a one-way analysis of variance (ANOVA) followed by the Dunnett post-hoc test. Prism 5 software (GraphPad Inc., La Jolla, CA, United States) was used for the statistical analysis.

## Results

### LL-37 inhibits the growth of pancreatic cancer cells

**FIGURE 1:**
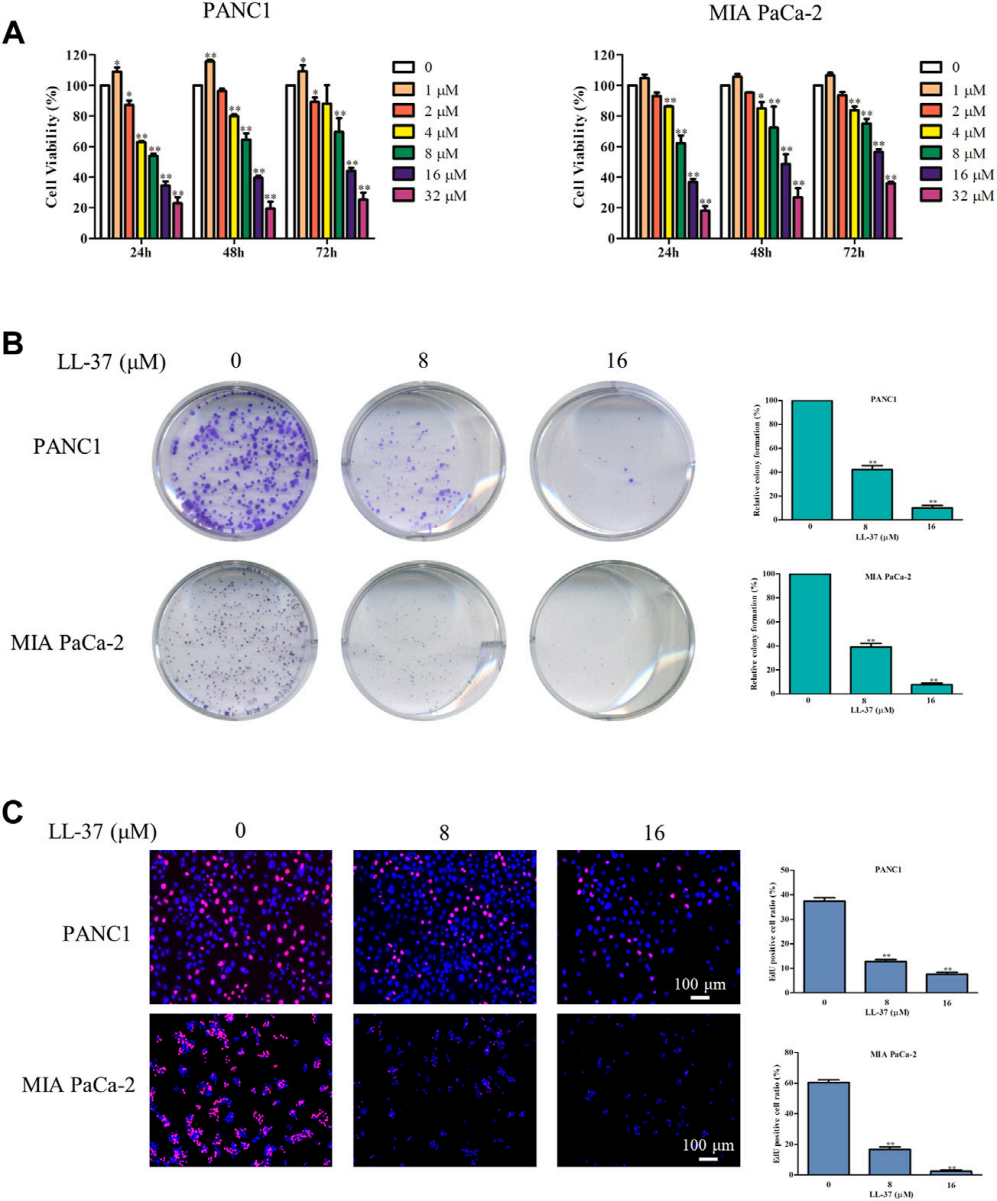
LL-37 inhibits the growth of pancreatic cancer cells. **(A)** The cell viability of human PANC1 and MIA PaCa-2 cells treated with various concentrations of LL-37 (1–32 μM) for 24, 48 or 72 h was determined by MTT assay. **(B)** Colony formation assay was used to evaluate the proliferation ability of PANC1 and MIA PaCa-2 cells. **(C)** The proliferation of PANC1 and MIA PaCa-2 cells was detected by EdU staining (red). DAPI (blue) stained the nuclei. Cells treated with DMSO were used as negative controls. The data of all drug treatment groups were normalized to the control group. Data are presented as mean ± SD of three independent experiments conducted in triplicate. *p < 0.05, **p < 0.01 *vs*. control (0 μM).

To confirm the inhibitory effect of LL-37 on pancreatic cancer cell growth, we treated PANC1 and MIA PaCa-2 cells with different concentrations of LL-37 (1–32 µM) for 24, 48 and 72 h before the testing with MTT assay. Results have showed that LL-37 at the concentration greater than 1 µM reduced the viability of both PANC1 and MIA PaCa-2 cells (Figure 1A) in a dose-dependent manner after treatment for 24 h. But no further growth-inhibitory effect was found with longer treatment time. Median growth-inhibitory concentrations (IC_50_) of LL-37 in PANC1 and MIA PaCa-2 cells after 24 h of treatments were found to be approximately 10.17 and 11.52 µM, respectively. Hence, the concentrations of 8 and 16 μM were selected for subsequent studies. Furthermore, the tumor colony formation was reduced after LL-37 treatment, indicating LL-37 inhibited the proliferative capacity of both PANC1 and MIA PaCa-2 pancreatic cancer cells (Figure 1B). This idea has been confirmed by the EdU staining assay whereas the ratio of EdU-positive cells was reduced after LL-37 treatment (Figure 1C). Taken together, LL-37 could inhibit the growth of pancreatic cancer cells.

### LL-37 induces the DNA damage response and cell cycle arrest in pancreatic cancer cells

**FIGURE 2:**
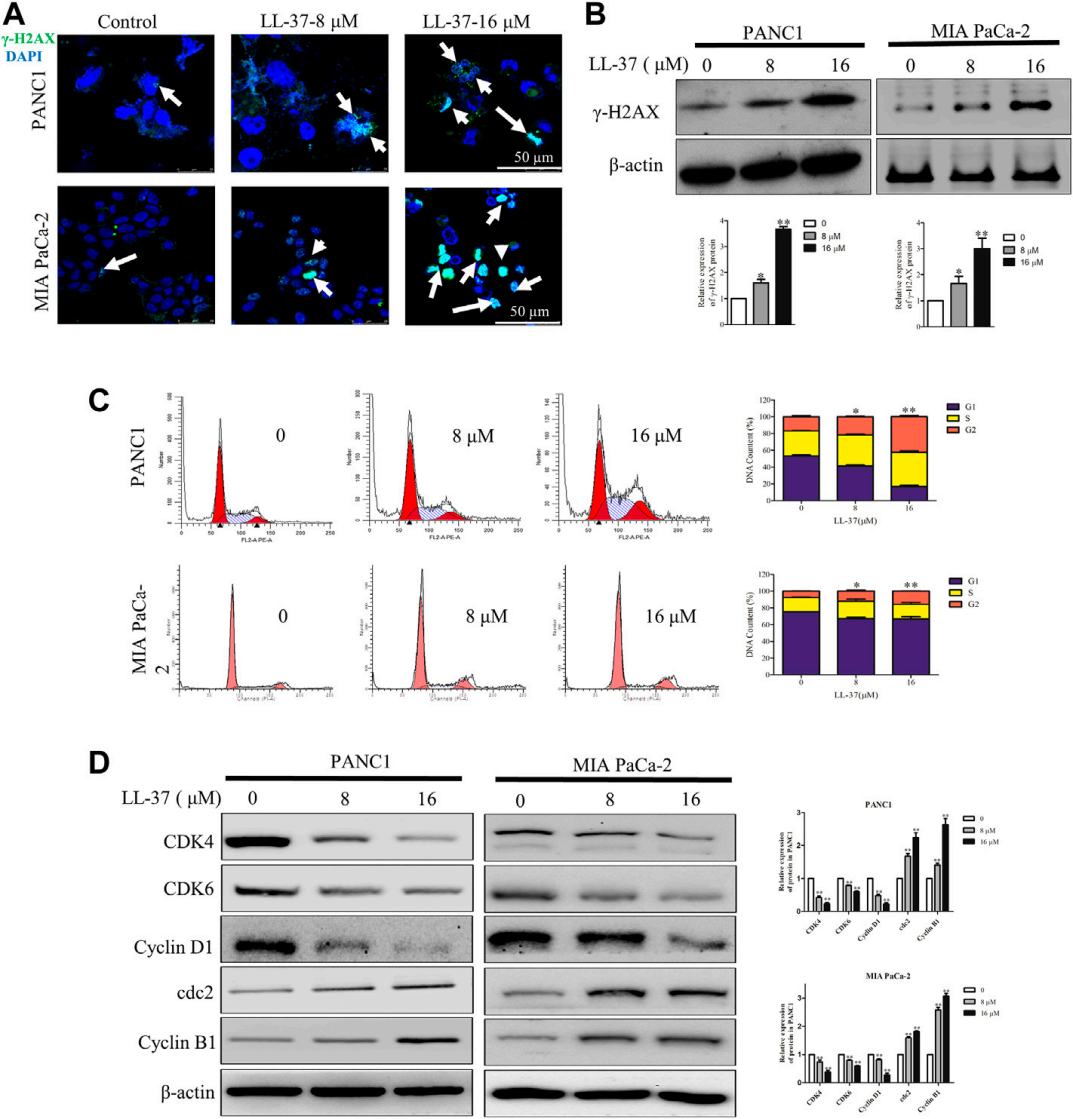
LL-37 induces DNA damage and cell cycle arrest in pancreatic cancer cells. LL-37 induced DNA damage by increasing γ-H2AX expression, as determined by immunofluorescence microscopy and western blotting **(A,B**), respectively after LL-37 (8 and 16 μM) treatment for 24 h. **(C)** The cell cycle distributions in PANC1 and MIA PaCa-2 cells were analyzed by flow cytometry after LL-37 treatment for 24 h. The data are presented as means ± SD. *p < 0.05, **p < 0.01 *vs*. control (0 μM) value in the G2 phase. **(D)** The expressions of cell cycle arrest related proteins were determined by western blotting after LL-37 treatment for 24 h. Non-arbitrary band density data relative to the housekeeping protein *ß*-actin are presented as mean ± SD, *p < 0.05, **p < 0.01 vs. control (0 μM).

To investigate whether the growth-inhibitory effect of LL-37 on pancreatic cancer cells may involve DNA damage, we examined the expression of γH2AX, a surrogate marker of DNA double-strand breaks using immunofluorescence microscopy. LL-37 increased formation of γH2AX foci in a dose-dependent manner in PANC1 and MIA PaCa-2 cells (Figure 2A), and this result was further confirmed by Western blotting (Figure 2B). Subsequent to the DNA damage response, LL-37 induced cell cycle arrest at G2 phases both in PANC1 cells and MIA PaCa-2 cells (Figure 2C). This was further confirmed as indicated by the decreased expression of CDK4, CDK6 and cyclin D1 as well as increased expression of cdc2 and cyclin B1 in PANC1 and MIA PaCa-2 cells after LL-37 treatment (Figure 2D). Taken together, our data indicate that LL-37 could induce DNA damage and subsequent cell cycle arrest in pancreatic cancer cells.

### LL-37 Decreases autophagy in pancreatic cancer cells through activation of the mTOR pathway

**FIGURE 3:**
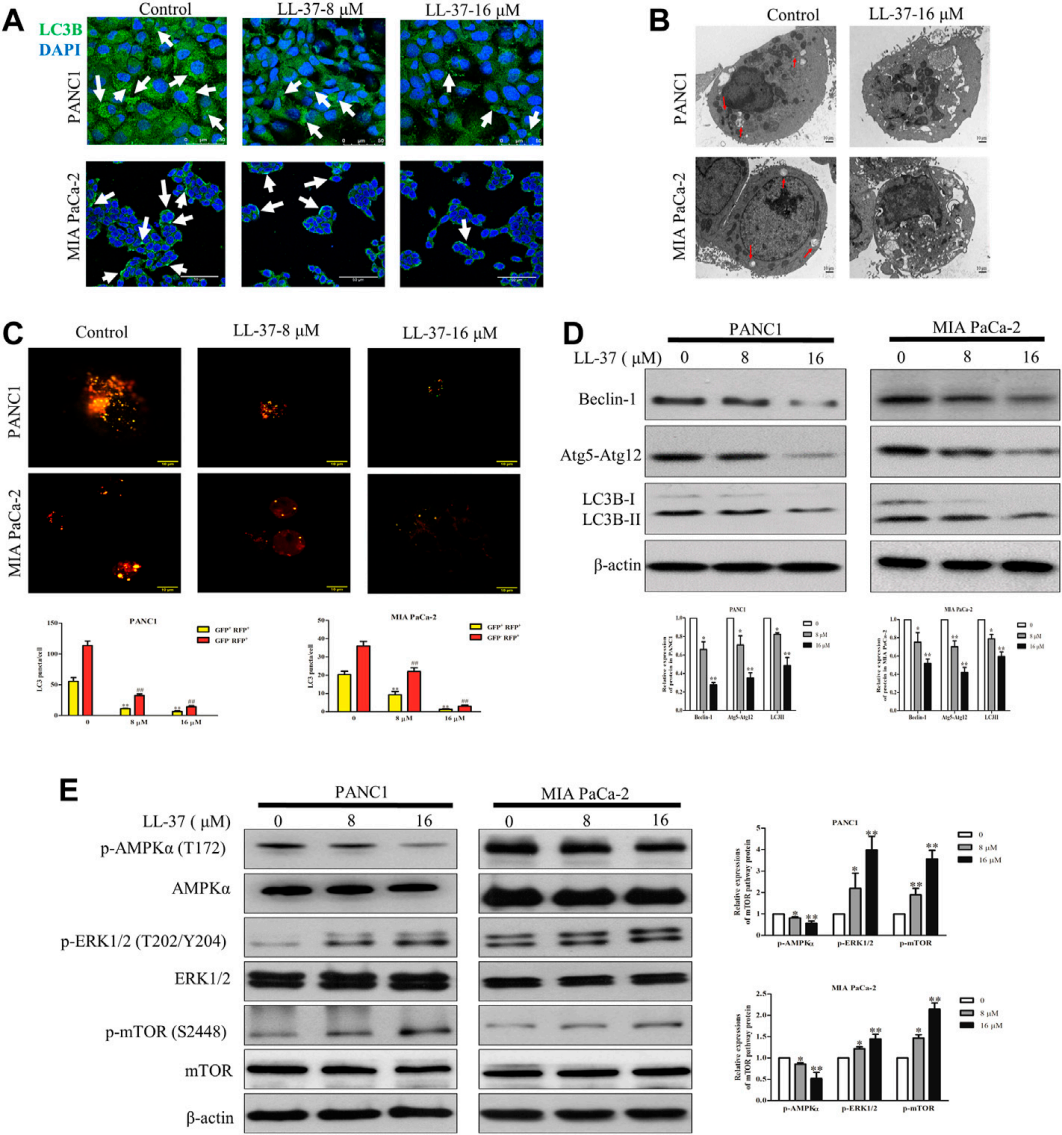
LL-37 inhibits autophagy in pancreatic cancer cells. **(A)** LL-37 (8 and 16 μM) decreased the formation of autophagosomes in pancreatic cancer cells as determined using immunofluorescence microscopy. **(B)** Autophagic vacuoles in PANC1 and MIA PaCa-2 cells treated with 16 μM LL-37 or DMSO were observed by TEM. The red arrow indicates autophagic vacuoles. **(C)** The autophagic flux in LL-37-treated PANC1 and MIA PaCa-2 cells was tested using the Autophagy Tandem Sensor RFP-GFP-LC3B. Representative images were photographed under a confocal microscope. The average numbers of yellow and red LC3B dots per cell are shown, **p < 0.01 *vs.* control of yellow dots, *##p* < 0.01 *vs*. control of red dots. **(D)** The levels of autophagy markers LC3II, Atg5–Atg12 and Beclin-1 in PANC1 and MIA PaCa-2 cells were measured by western blotting after treatment with LL-37 for 24 h. **(E)** The mTOR pathway involved in the inhibitory effect of LL-37 on autophagy was determined by western bloting. Cells treated with DMSO were used as negative controls. Three independent experiments were each performed in triplicate. Representative protein bands are shown for comparison. Non-arbitrary band density data relative to the housekeeping protein *ß*-actin are presented as mean ± SD, *p < 0.05, **p < 0.01 *vs*. control (0 μM).

To evaluate the effect of LL-37 on the autophagy process, LC3B level in pancreatic cancer cells was determined using immunofluorescence confocal microscopy after treatment of LL-37 for 24 h. Results have shown that LL-37 decreased the formation of LC3B puncta in PANC1 and MIA PaCa-2 cells (Figure 3A), which indicates decreased formation of autophagosomes. Direct evidence of drug effect on autophagy was acquired from experiments using TEM, of which LL-37 treatment led to the formation of fewer autophagic vacuoles containing intracellular aggregates, damaged organelles and empty vacuoles (Figure 3B). To provide more evidence that LL-37 could inhibit autophagic flux in pancreatic cancer cells, we transiently transferred tandem RFP-GFP-LC3B into PANC1 and MIA PaCa-2 cells. Expression of tandem RFP-GFP-LC3B resulted in both red and green fluorescence. The red puncta that overlayed with the green puncta (merged as yellow) were indicators of autophagosomes (neutral pH) that are not fused with acidic lysosomes, which implicates the formation of autophagosomes. Nevertheless, the solely red puncta were indicative of autolysosomes, which indicates the process of autophagy flux. Our results have showed that both yellow and red puncta were decreased after LL-37 treatment (Figure 3C), which suggests that LL-37 suppresses autophagy flux. In addition, the protein level of autophagy-related protein markers LC3B-II, Beclin-1 and Atg5-Atg12 was reduced by LL-37 (Figure 3D). mTOR is a critical regulator of autophagy in cancer cells and is usually regulated by the PI3K-Akt, Ras-Raf-1-MEK1/2-ERK1/2 and AMPK pathways (Meley et al., 2006; Pópulo et al., 2012). To explore the effects of LL-37 on autophagy-related signal transduction pathways, we have evaluated the protein expression of various signaling upstream regulators of autophagy. LL-37 induced the phosphorylation of mTOR in both PANC1 cells and MIA PaCa-2 cells, with inactivation of AMPKα and activation of ERK1/2 in both cell types (Figure 3E). Taken together, these data suggest that LL-37 decreased the autophagy levels in pancreatic cancer cells involved the activation of the mTOR pathway.

### LL-37 increases intracellular ROS production due to its inhibition of autophagy

**FIGURE 4:**
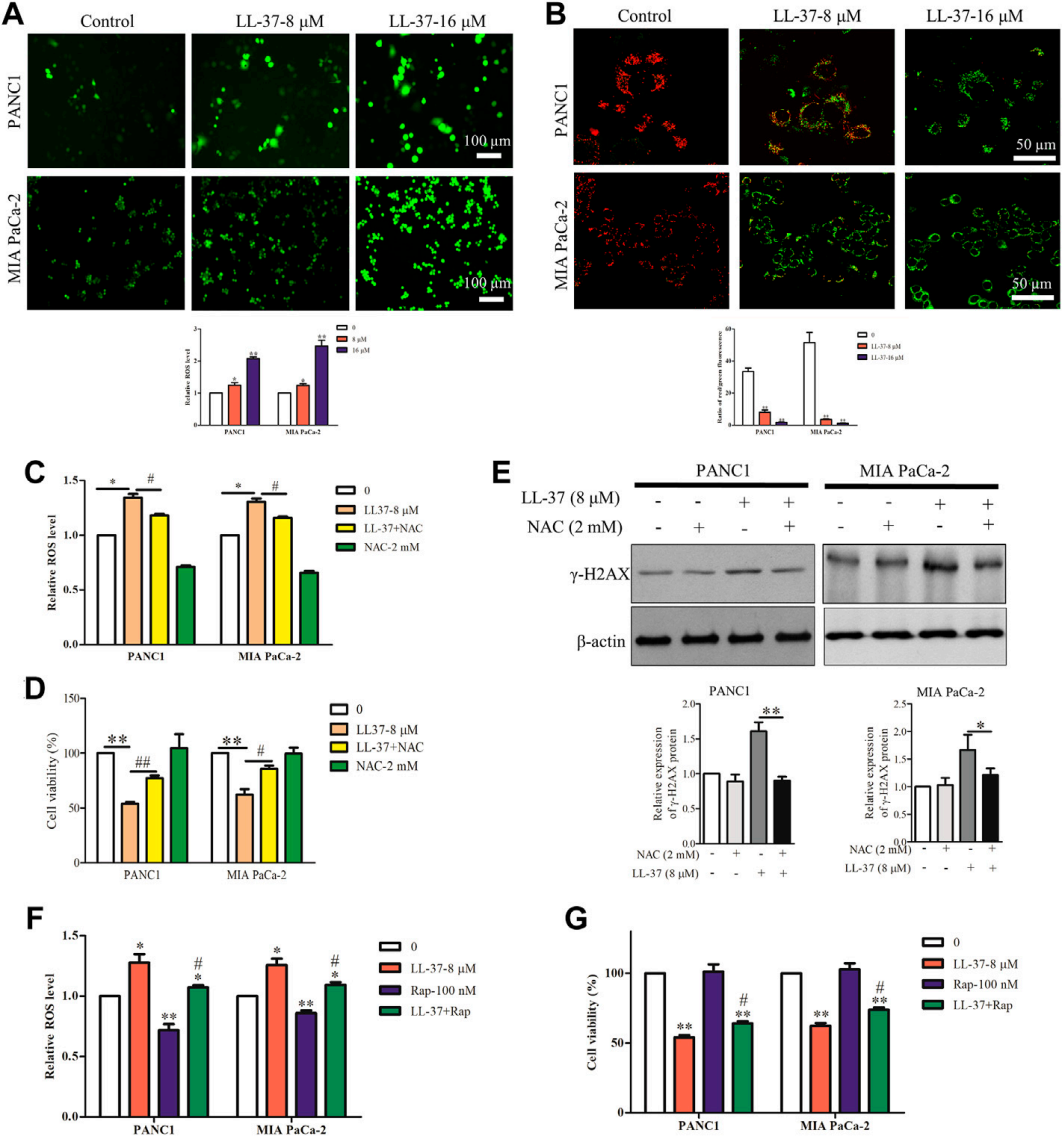
LL-37 increases the intracellular production of reactive oxygen species (ROS) in pancreatic cancer cells. **(A)** ROS were detected using the fluorescence dye CM-H2DCFDA and immunofluorescence microscopy. ROS fluorescence was measured using a SpectraMax Plate Reader (Molecular Devices) with excitation/emission wavelengths of 495/520 nm. Data are presented as mean ± SD of three independent experiments conducted in triplicate. *p < 0.05, **p < 0.01 *vs*. control (0 μM). **(B)** Effect of LL-37 on the MMP in PANC1 and MIA PaCa-2 cells was measured using JC-1 staining method. Representative images were photographed under a confocal microscope. The red/green fluorescence ratio was quantified by the Image J. **p < 0.01 *vs.* control (0 μM). **(C)** The ROS production of pancreatic cancer cells treated with LL-37 in the presence and absence of a ROS scavenger NAC (2 mM) was determined. *p < 0.05 *vs*. control; ^#^ *p* < 0.05 *vs*. LL-37 (8 µM). **(D)** The viability of pancreatic cancer cells treated with LL-37 in the presence and absence of a ROS scavenger NAC was determined using an MTT assay. **p < 0.01 *vs*. control; ^#^ *p* < 0.05, ^##^ *p* < 0.01 *vs*. LL-37 (8 µM). **(E)** The expression of γ-H2AX in PANC1 and MIA PaCa-2 cells treated with LL-37 in the presence and absence of NAC was determined by western blotting. Non-arbitrary band density data relative to that of the housekeeping protein *ß*-actin are presented as mean ± SD, *p < 0.05, **p < 0.01 *vs*. LL-37 (8 µM). The ROS production **(F)** and cell viability **(G)** were determined in pancreatic cancer cells treated with LL-37 in the presence and absence of an autophagy inducer (rapamycin; 100 nM), respectively. *p < 0.05, **p < 0.01 *vs*. control; ^#^ *p* < 0.05 *vs.* LL-37 (8 µM).

The loss of autophagy can cause accumulation of damaged mitochondria and the oxidative protein folding machinery, which promotes ROS production and activated DNA damage response (Mathew et al., 2009). Immunofluorescence analysis has revealed an increased ROS production after LL-37 treatment (Figure 4A), and this result was further confirmed quantitatively (Figure 4A). Excessive ROS formation usually induces a decline in MMP and impairs mitochondrial function that eventually causes cell death. To measure the MMP in the pancreatic cancer cells after LL-37 treatment, the JC-1 MMP probe was employed. Results have showed that LL-37 decreased the ratio of red/green fluorescence in pancreatic cancer cells (Figure 4B), which indicates the MMP collapses and the potential induction of cell death. To determine whether ROS mediated the growth-inhibitory effect of LL-37 on pancreatic cancer cells, we added the ROS scavenger NAC to the cells treated with LL-37. Results have showed that both LL-37-induced ROS accumulation and growth inhibition were reversed by drug treatment through combining with NAC (Figures 4C,D). Further analysis has revealed that treatment with NAC reversed the effects of LL-37 on the expression of γH2AX in both PANC1 and MIA PaCa-2 cells (Figure 4E). To explore whether LL-37-induced suppression of autophagy contributed to the accumulation of ROS and cell growth inhibition, we measured ROS production and the viability of pancreatic cancer cells in the presence or absence of the autophagy inducer rapamycin. Results have showed that rapamycin attenuated LL-37-induced ROS production (Figure 4F) and weakened LL-37-induced growth inhibition of pancreatic cancer cells (Figure 4G). Taken together, these data suggest that LL-37-induced inhibition of autophagy results in ROS accumulation and subsequent growth inhibition of pancreatic cancer cells.

### LL-37 inhibits pancreatic tumor growth *in vivo*

**FIGURE 5:**
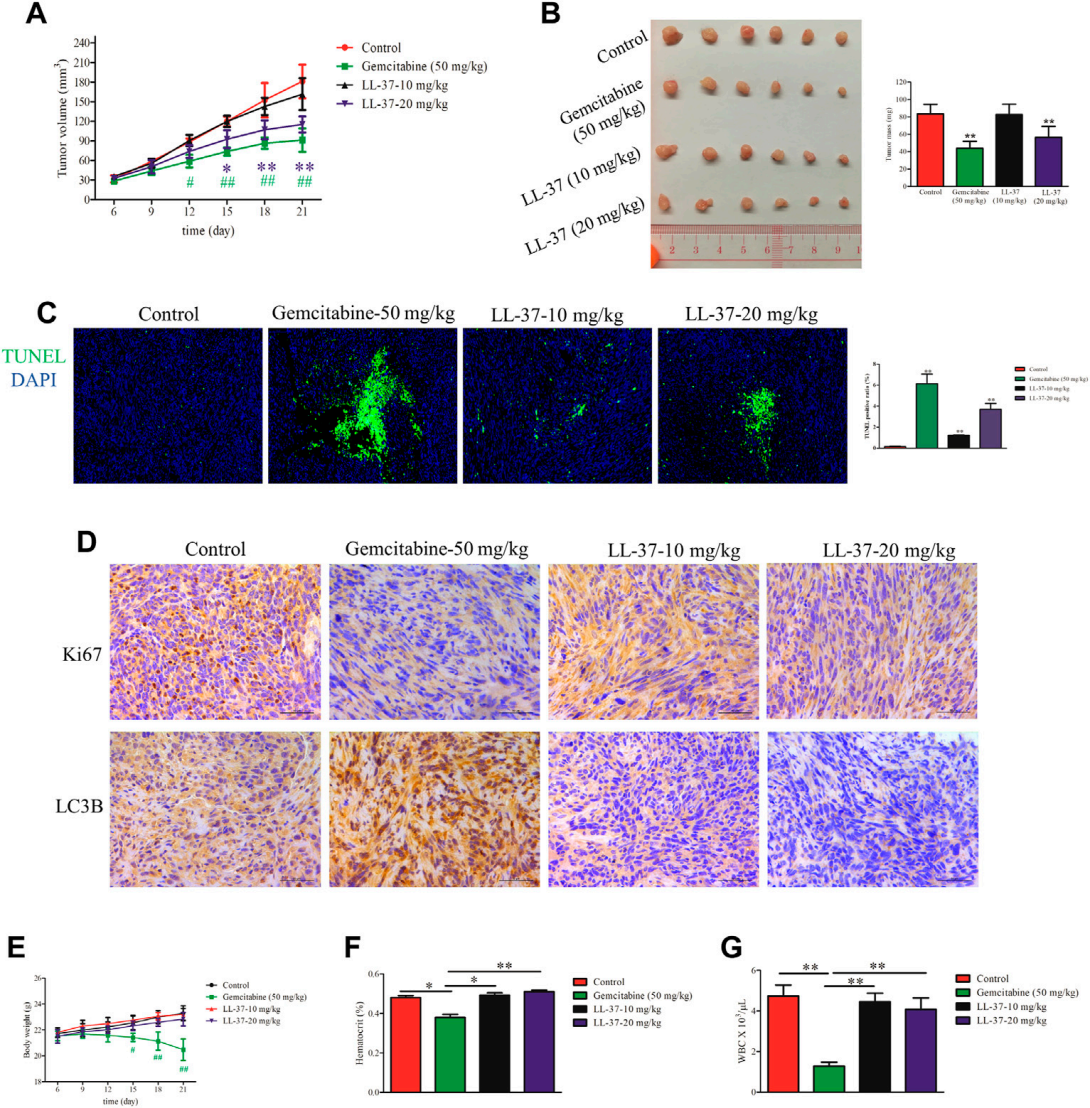
LL-37 inhibited the growth of pancreatic tumors *in vivo*. **(A)** The subcutaneous tumor diameters were measured every 3 days, and the tumor volumes were calculated using the formula: (short diameter)^2^ × (long diameter) × 0.5. *p < 0.05, **p < 0.01 for LL-37 20 mg/kg *vs.* control group; ^#^ *p* < 0.05, ^##^ *p* < 0.01 for gemcitabine *vs*. control group. **(B)** The tumor weights in the control group, positive drug group (gemcitabine 50 mg/kg), low-dose LL-37 (10 mg/kg) group and high-dose LL-37 (20 mg/kg) group. **p < 0.01 *vs*. control group. **(C)** Tumor tissues from mice in different groups were subjected to a TUNEL apoptosis assay. **(D)** Immunohistochemistry analysis to detect Ki67 and LC3Ⅱ expression in the tumor tissues of mice in different groups (scale bar = 50 μm, magnification × 400). **(E)** The body weights were measured every 3 days ^#^ *p* < 0.05, ^##^ *p* < 0.01 gemcitabine *vs*. control group. After treatment for 14 days, the hematocrit **(F)** and white blood cells **(G)** in the peripheral blood were evaluated. *p < 0.05, **p < 0.01 *vs*. gemcitabine group.

A murine subcutaneous tumor xenograft model was used to determine the growth-inhibitory effects of LL-37 *in vivo*. The tumor volumes in mice treated with gemcitabine (positive control) or high-dose LL-37 (20 mg/kg) were significantly smaller than that in the control group after 12 and 15 days post-tumor inoculation, respectively (Figure 5A). As shown in Figure 5B, high-dose of LL-37 at 20 mg/kg caused 42% reduction in tumor growth with respect to the no treatment control, whereas low-dose of LL-37 at 10 mg/kg produced insignificant growth-inhibitory effect. The analysis of TUNEL-stained tumor tissues showed that LL-37 treatment increased the population of apoptotic cells in the tumor tissue (Figure 5C). IHC analysis has further revealed the decreased expression of Ki67 in the nucleus after LL-37 treatment, which indicates its anti-proliferative effect on pancreatic tumor cells (Figure 5D). The IHC analysis of LC3B showed that gemcitabine (positive control) treatment induced higher autophagy level, which could partly explain the presence of drug resistance (Figure 5D). Compared with gemcitabine, LL-37 induced fewer adverse effects in the mice, as indicated by a stable body weight, white blood cell count and hematocrit (Figures 5E–G, respectively). Taken together, our data suggest that high-dose LL-37 inhibits pancreatic tumor growth *in vivo* while inducing fewer side effects than gemcitabine.

### LL-37 reprograms the immune cells in the tumor microenvironment

**FIGURE 6:**
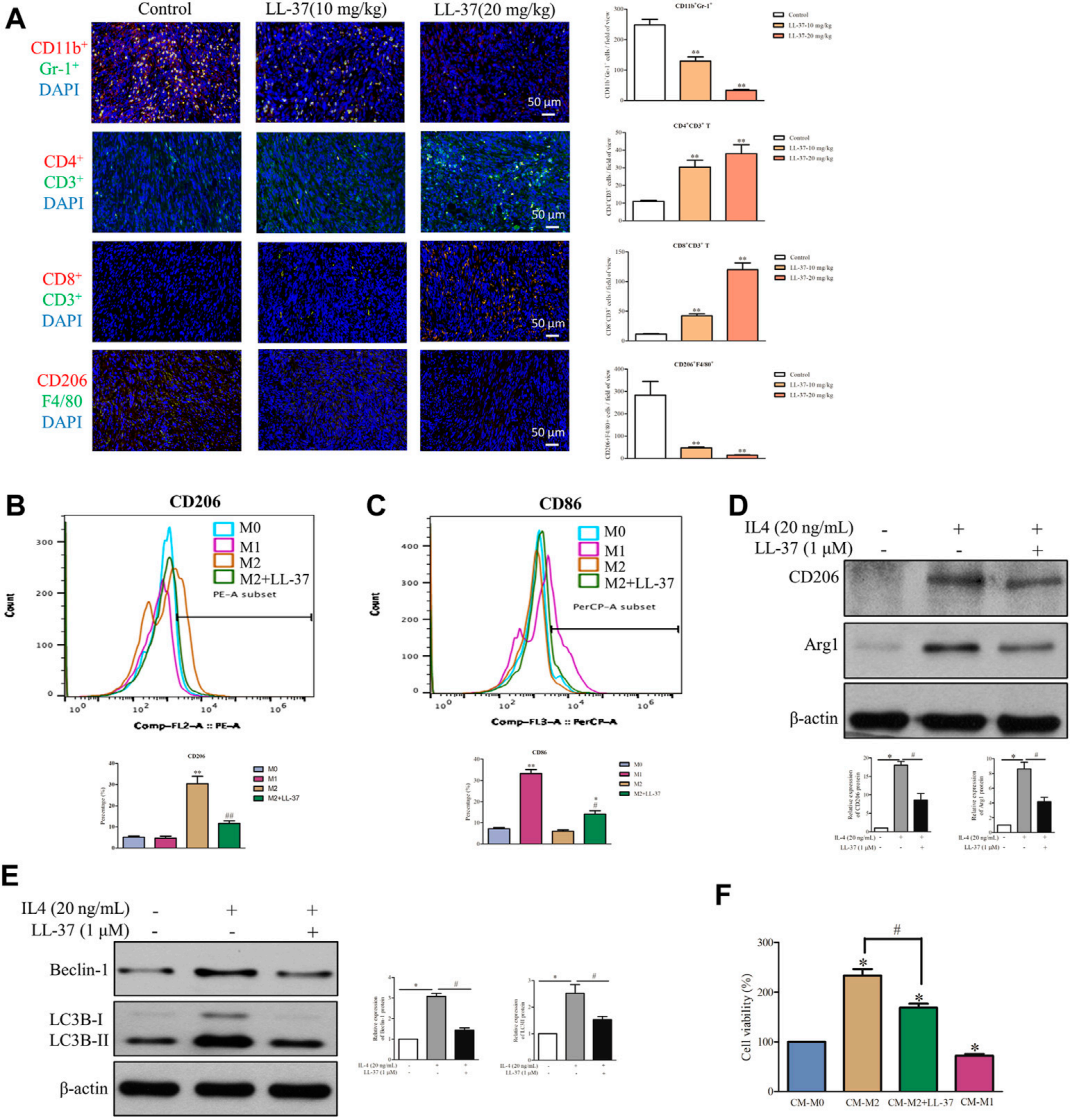
LL-37 reprogrammed the immune cells in the tumor microenvironment. **(A)** A dual-immunofluorescence analysis was used to identify the myeloid-derived suppressor cells (MDSCs), CD4^+^ T cells, CD8^+^ T cells and M2 macrophages in the tumor tissues (scale bar = 50 μM, 400× magnification). The positively stained cells were quantified using Image-pro plus 6.0. **p < 0.01 *vs*. Control group. The effect of LL-37 on the polarization of macrophages was determined using a flow cytometer. F4/80 is the common marker of macrophages cell. **(B)** CD206 is a specific surface marker for M2 macrophages. **(C)** CD86 is a specific surface marker for M1 macrophages. The percentage of CD206 and CD86 expression on F4/80 macrophages were quantified. *p < 0.05, **p < 0.01 *vs.* M0 group, ^#^ *p* < 0.05, ^# #^ *p* < 0.01 *vs*. M2 group. The effect of LL-37 on the expression of the M2 macrophage markers **(D)** and autophagy markers **(E)** measured using western blot in RAW264.7 cells treated with IL-4. Non-arbitrary band density data relative to those of the housekeeping protein *ß*-actin are presented as mean ± SD, *p < 0.01 for IL-4 treatment *vs*. control, ^#^ *p* < 0.01 for LL-37 + IL-4 treatment *vs*. IL-4 treatment. **(F)** Cell viability of PAN02 cultured with conditioned medium from M0 macrophages (CM-M0), M1 macrophages (CM-M1), M2 macrophages (CM-M2) and M2 macrophages + LL-37 (CM-M2+LL-37). CM-M1 is positive control. Data are presented as the means ± SD of three independent experiments each conducted in triplicate. *p < 0.01 for CM-M2 *vs*. CM-M0, ^#^ *p* < 0.01 for CM-M2+LL-37 *vs*. CM-M2.

Accumulating evidence shows that pancreatic cancer induces an immunosuppressive microenvironment, which lead to the suppression of immune effectors and response to therapies (Chen et al., 2005). In this study, a dual-immunofluorescence analysis was performed to study the effects of LL-37 on the tumor immune microenvironment. LL-37 treatment appeared to reduce the immunosuppressive cells of MDSCs (CD11b^+^Gr-1^+^) and M2 macrophages (CD206^+^F4/80^+^) and to increase the anti-cancer effectors CD4^+^T (CD4^+^CD3^+^) and CD8^+^T (CD8^+^CD3^+^) cells (Figure 6A). To study the effect of LL-37 on the polarization of macrophages, we measured the subtypes of RAW264.7 cells after LL-37 treatment using flow cytometry. Results have showed that LL-37 decreased CD206 expression and increased CD86 expression in IL-4 induced M2 macrophages, which indicates that LL-37 could impair M2 polarization and further reversed M2 macrophages to M1 phenotype (Figures 6B,C). LL-37 induced impairment of M2 macrophage polarization was also confirmed by reduced protein expression of the IL-4-induced M2 markers CD206 and Arg1 (Figure 6D). Further study showed that LL-37 reduced IL-4-induced autophagy in macrophage cells with decreased expression of Beclin-1 and LC3B-II (Figure 6E). To explore the effect of reduced M2-polarized macrophage populations on the growth of pancreatic cancer, we derived conditioned medium from LL-37-treated M2 macrophages. The results showed that co-culture with M2 macrophage increased the growth of PAN02 cells, which was prevented by LL-37 treatment (Figure 6F). Based on these findings, we propose that LL-37 may also exert anti-tumorigenic effects in pancreatic cancer by reprogramming the immune system in the tumor microenvironment.

## Discussion

LL-37, being the only cathelicidin family member expressed in humans, exerts differential effects in various forms of cancers. When compared to normal tissues, overexpression of hCAP-18/LL-37 mRNA and protein was observed in the stroma of ovarian cancer, lung cancer, breast cancer, prostate cancer, malignant melanoma, and skin squamous cell carcinoma. However, in colon cancer, gastric cancer, hematologic malignancy and oral squamous cell carcinoma, LL-37 expression levels are downregulated compared to the normal tissues. Therefore, tumor-derived LL-37 may act either as a pro-tumorigenic or anti-cancer agent depending on tumor biology. For exogenous recombinant LL-37, it seems to be concentration-dependent. As we have discussed above, at concentration of 5 μg/ml (1.1 µM), LL-37 exerts pro-tumorigenic effects in many cancer types. At higher concentrations, however, LL-37 induces anti-cancer effects in several other cancer types. Similar observation was also illustrated in our study, whereas LL-37 promoted the growth of PANC1 cells at the concentration of 1 µM, while it inhibited cell viability when the concentration becomes higher than 2 µM. For the first time, our findings indicate that LL-37 can inhibit the growth of pancreatic cancer cells by suppressing autophagy. As mentioned before, autophagy can be regulated by mTOR in cancer cells. This kinase assembles into two distinct complexes, mTOR complex 1 (mTORC1) and mTOR complex 2 (mTORC2). Although both complexes are implicated in growth factor sensing, mTORC1 is generally associated with cell proliferation and cancer progression when dysregulated (Pópulo et al., 2012). Three pivotal mTORC1 signaling pathways are involved in autophagy: the PI3K-Akt and Ras-Raf-1-MEK1/2-ERK1/2 pathways, which activate mTORC1, and the AMPK pathway, which can inhibit mTORC1 (Meley et al., 2006). Under energetic stressful conditions, the activation of upstream autophagy-associated genes AMPK can stimulate ULK1 and inhibit mTOR signaling, resulting in increased autophagy. Previous studies reported that AMPK facilitates tumor cell growth, whereas the absence of AMPK drastically attenuates tumorigenicity *in vivo* (Mathew et al., 2009; Walczak and Martens 2013). Elevated AMPK expression increases the aggressiveness of pancreatic cancer cells and suggests a poor prognosis. The activation of AMPK enhances the stem-like properties of pancreatic cancer cells, whereas the inhibition of AMPK drastically impairs the proliferative and invasive capacities of pancreatic tumor cells (Shen et al., 2018). Activation of AMPK also suppresses the activation of mTORC1 and leads to the induction of autophagic flux in pancreatic cancer cells (Xu et al., 2019). In addition, excessive ERK1/2 activation was proven to promote autophagic cell death (Wang et al., 2009) and stimulate mTOR activation (Carriere et al., 2011). So far, there is almost no report about the influence of LL-37 on the autophagy in cancer cells. Although FK-16, a shorter fragment of LL-37, induced autophagy in colon cancer cells, it was also proved to augment AIF−/EndoG-dependent apoptosis in colon cancer cells when autophagy was inhibited (Ren et al., 2013). In the present study, we first demonstrated that LL-37-induced autophagy suppression through mTOR activation leads to mitochondrial membrane potential decline and ROS accumulation as well as the resulting DNA damage in pancreatic cancer cells.

Most human PDACs exhibit an elevated basal level of autophagy, which is accompanied by the accumulation of ROS during the development and progression of pancreatic cancer (Jeon et al., 2012; Laderoute et al., 2014). In turn, the loss of autophagy can cause an accumulation of damaged mitochondria and the oxidative protein folding machinery, which would promote the production of ROS (Mathew et al., 2009). Consistent with those earlier findings, our results have demonstrated that LL-37 increased ROS accumulation and the subsequent mitochondrial dysfunction by inhibiting autophagy in pancreatic cancer cells, which results in DNA damage and cell death. In a previous study of PDAC, autophagy was shown to enable growth by preventing cells from accumulating genotoxic levels of ROS and sustaining oxidative phosphorylation by providing bioenergetic intermediates (Yang and Kimmelman 2011). However, it is worth mentioning that ROS act as a “double-edged sword” on tumor development, as both the inducer and inhibitor of ROS that may alter cancer cell death (including pancreatic cancer) by disrupting the redox balance (Marchi et al., 2012). Mildly or moderately elevated levels of ROS promote the initiation of carcinogenesis and the malignant transformation of cells, whereas excessive ROS evoke irreversible oxidative damage and trigger programmed cell death (Fruehauf and Meyskens, 2007). Excessive ROS-triggered DNA damage was demonstrated to induce cancer cell death in various cancer types including pancreatic cancer (Yang et al., 2011). Genetic or pharmacological inhibition of autophagy may provoke ROS formation and DNA damage that could significantly inhibit pancreatic cancer cell growth (Yang et al., 2011). Here, we have shown a coherent result that LL-37-induced ROS accumulation resulted in DNA damage and cell cycle arrest. Similarly, LL-37 treatment was shown to induce cell cycle arrest by increasing the expression of p21^Waf1/Cip1^ in gastric cancer cells and to induce-large-scale induction of DNA fragmentation in colon cancer cells (Mader et al., 2009; Kuroda et al., 2017).

In patients with pancreatic cancer, immune dysfunction is observed in the immunosuppressive tumor microenvironment, which inhibits the activation or functions of immune effectors (Chen et al., 2005). Decreased circulating CD4^+^ and CD8^+^ T cell populations are observed in patients with pancreatic cancer when compared with healthy individuals (Bang et al., 2006). Evidence suggests that inhibition of autophagy would impair tumor metabolism and alter tumor microenvironmental compartments, which could dampen the antitumor immune response. For example, aging T lymphocytes exhibit a steady decrease in autophagy (Phadwal et al., 2012). Furthermore, autophagy played a key role in tumor-specific CD8^+^ T cell priming (Uhl et al., 2009). More studies, however, indicate that autophagy inhibition does not impair T cell function in tumor models such as melanoma and breast cancer (Starobinets et al., 2016). Treatment with HCQ enhanced the cytotoxic T-cell activity during hypoxic stress (Noman et al., 2011). Another report also demonstrated that lysosomes limited the CD8^+^ T-cell killing activity in melanoma (Khazen et al., 2016). Inhibition of autophagy in response to mutated Beclin1 augmented the infiltration of T cells into the immune microenvironment (Mgrditchian et al., 2017). CD8^+^ T cells were proven to accumulate around PDAC cells with low levels of autophagy flux but avoided cells with high autophagy flux (Yamamoto et al., 2020). In our study, LL-37 treatment reduced MDSC population and increased CD4^+^ and CD8^+^ T cell populations in the tumor microenvironment, and this shift may have contributed to the observed anti-tumor effects. This finding is consistent with a recent study in which the tumor-specific inhibition of autophagy caused increased CD8^+^ T cell proliferation, activation and cytotoxicity against pancreatic cancer cells (Yamamoto et al., 2020). Another study has determined that LL-37 frequently induces CD4^+^ and CD8^+^ T cell proliferation and activation (Lande et al., 2014). Furthermore, the exposure of dendritic cells to LL-37 increased the migration of CD8^+^ T cells into established squamous cell carcinomas in mice, resulting in regression of the tumor (Findlay et al., 2019). Nevertheless, recent studies have demonstrated that treatment with lysosomal inhibitors such as CQ can enhance antitumor T-cell immunity by switching macrophage polarization from tumor-associated macrophages M2 to tumor-killing (and pro-inflammatory) M1 phenotype (Chen et al., 2018). This switch would enable tumor cell killing by cytotoxic T cells without affecting dendritic cells (Noman et al., 2011; Starobinets et al., 2016). In another study, autophagy inhibition was determined to decrease the growth of pancreatic tumor in the dominant-negative Atg4b mouse model, which is also involved in the macrophage-mediated mechanisms (Yang et al., 2018). We have recently demonstrated that the increased polarization of M2 macrophages promoted the progression of pancreatic cancer (Zhang et al., 2020). In this study, we have evaluated the effect of LL-37 on macrophage polarization both *in vitro* and *in vivo* and revealed that M2 macrophage polarization was inhibited in the pancreatic tumor microenvironment, with significant contribution to growth inhibition of solid tumors. Furthermore, this phenomenon was also accompanied with LL-37-induced autophagy inhibition in IL-4 treated macrophage cells. In preclinical studies, the intratumorally injections of recombinant LL-37 stimulate the innate immune system by activation of plasmacytoid dendritic cells (Dolkar et al., 2018). These cells can induce and maintain antitumor immune responses that mediate tumor destruction (Hargadon, 2017). Taken together, our data concerning the role of autophagy in tumor-host interactions and tumor immunity suggest that other than targeting tumor cells, the promotion of autophagy inhibition in many other non-malignant cell types could be equally important.

## Conclusion

In conclusion, we report for the first time that treatment with the unique peptide drug LL-37 could inhibit the growth of pancreatic cancer cells through inhibition of autophagy and reprogramming of the tumor immune microenvironment. These findings provide important insights on the anti-cancer mechanism of LL-37 in the treatment of pancreatic cancer by exerting diversified and target-specific modulating modalities.

## Data Availability

The raw data supporting the conclusion of this article will be made available by the authors, without undue reservation.

## Abbreviations

ANOVA, One-way analysis of variance; CQ, Chloroquine; DAPI, 4′,6-diamidino-2-phenylindole; DMSO, Dimethyl sulfoxide; DMEM Dulbecco’s modified Eagle’s medium; EdU 5-Ethynyl-2’-deoxyuridine; γH2AX Ser139-phosphorylated histone H2AX; IHC, Immunohistochemical; ROS Reactive oxygen species; PDAC Pancreatic ductal adenocarcinoma PDAC; HCQ Hydroxychloroquine; MDSCs Myeloid-derived suppressor cells; MMP Mitochondrial Membrane Potential; MTT, 3-(4,5-dimethylthiazol-2-yl)-2,5-diphenyltetrazolium bromide; NAC, N-acetyl cysteine NAC; SD, Standard deviations.
